# Case Report: Presenting as optic neuritis—a biopsy-proven IgG4 anti-NF155–positive combined central and peripheral demyelination syndrome

**DOI:** 10.3389/fimmu.2026.1793527

**Published:** 2026-05-22

**Authors:** Yujing Peng, Xiaonan Wang, Weijiao Zhang, Ran Li, Jingting Peng, Jiawei Wang, Hanqiu Jiang

**Affiliations:** Department of Neurology, Beijing Tongren Hospital, Capital Medical University, Beijing, China

**Keywords:** anti-neurofascin 155, case report, combined central and peripheral demyelination, histopathological examination, optic neuritis

## Abstract

Combined central and peripheral demyelination (CCPD) is a condition characterized by concurrent demyelination in both the central and peripheral nervous systems. To date, cases of CCPD presenting with isolated optic neuritis as the initial manifestation and positive for anti-neurofascin 155 (anti-NF155) antibodies are exceedingly rare. We herein report a case of a young male positive for anti-NF155 antibodies who developed severe optic neuritis and was subsequently diagnosed with CCPD via histopathological examination. This case highlights that CCPD may develop in patients presenting with optic neuritis as the initial manifestation, and underscores the critical importance of anti-NF155 antibody testing when concomitant peripheral neuropathy is identified.

## 1 Introduction

Combined central and peripheral demyelination (CCPD) is a rare neuroimmune disorder characterized by the co-occurrence of demyelinating lesions in both the central nervous system (CNS) and peripheral nervous system (PNS) (1). Its clinical manifestations are highly heterogeneous, ranging from isolated peripheral neuropathy to complex presentations involving optic nerve dysfunction, myelopathy, and encephalopathy (2, 3). Notably, optic nerve involvement is frequent but often subclinical, detectable only by abnormalities in visual-evoked potentials (VEP) in a substantial proportion of patients (4). This underscores that visual pathway impairment can be a prominent and, in some cases, the inaugural manifestation of CCPD. The pathogenesis of CCPD is increasingly linked to specific autoantibodies, particularly IgG4 subclass antibodies targeting paranodal proteins such as neurofascin-155 (NF155) (1, 5). Anti-NF155 antibody-positive (NF155^+^) CCPD represents a distinct clinicopathological phenotype, frequently associated with a poor response to intravenous immunoglobulin (IVIg), potential nerve root hypertrophy on imaging, and a high prevalence of subclinical optic nerve demyelination (6, 7). The shared expression of NF155 in CNS oligodendrocytes and PNS Schwann cells provides a mechanistic basis for the simultaneous demyelination in both compartments (8). Current management strategies for NF155^+^ CCPD with visual involvement necessitate aggressive and timely immunomodulation to prevent irreversible optic nerve damage (9). However, patients with anti-NF155 antibodies often respond poorly to first-line chronic inflammatory demyelinating polyneuropathy (CIDP) treatments such as IVIg (6). In contrast, corticosteroids and B-cell depleting therapy with rituximab have demonstrated higher efficacy in this subgroup (10, 11). Critically, the therapeutic course is often complicated by steroid dependence and a significant risk of relapse upon tapering or discontinuation, highlighting an urgent need for effective steroid-sparing maintenance regimens to secure long-term remission (2). Herein, we report a case of NF155^+^ CCPD in a young man who presented with severe, isolated optic neuritis, later confirmed by sural nerve biopsy and serological testing. His clinical course was marked by steroid dependence, relapse upon treatment discontinuation, and ultimately sustained remission achieved with corticosteroid pulse therapy combined with rituximab.

## 2 Case description

A 21-year-old man developed blurred vision in his right eye, starting from the temporal field and gradually progressing to involve the entire visual field in April 2022. One month later, he began experiencing weakness in both lower limbs, particularly in his feet, resulting in significant foot drop. Two months later, his left eye developed analogous symptoms, and he progressively lost the ability to walk independently. Subsequently, he also developed difficulty maintaining a stable grip on objects. The patient initially received treatment at the ophthalmology department of a local hospital. At that time, his visual acuity was measured at 0.3 in the right eye and 0.4 in the left eye. Humphrey visual field testing revealed bilateral visual field defects in the outer superior quadrants (**Supplementary Figure 1A**). VEP demonstrated significantly reduced P100 amplitudes bilaterally. Electromyography (EMG) findings were consistent with demyelinating sensorimotor polyneuropathy. In light of the electrophysiological evidence of demyelinating polyneuropathy, he underwent intravenous methylprednisolone therapy (1 g/day for 3 days, followed by 500 mg/day for 3 days), subsequently transitioning to oral prednisolone at 60 mg/day with a weekly taper of 5 mg. Despite this treatment regimen, the etiology of his visual impairment remained undetermined. While his lower limb weakness showed slight improvement, there was no significant recovery in bilateral visual impairment or upper limb weakness. He was subsequently referred to our neurology department three months after symptom onset, while maintaining a prednisolone dosage of 45 mg/day.

There was no history of recent international travel, new medication use, or toxic exposure. Family history was unremarkable for neurological or autoimmune disorders.

Neurological examination revealed isocoric pupils (3 mm bilaterally) with prompt light reflexes and no relative afferent pupillary defect (RAPD). Extraocular movements were intact. The patient exhibited mild distal limb weakness, diffusely hyporeflexic tendon reflexes, ataxic gait, and intention tremor. Both heel-to-shin and finger-to-nose tests were abnormal due to dysmetria. No other cranial nerve deficits were identified. The remainder of the physical examination was unremarkable.

**Figure 1:**
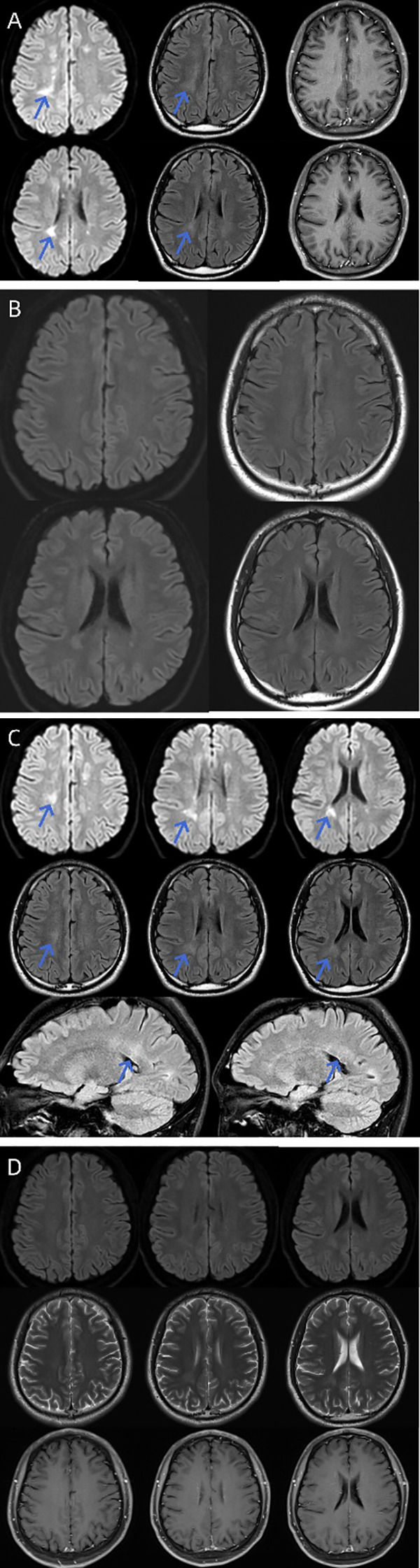
Serial brain MRI demonstrating the dynamic evolution of intracranial demyelinating lesions. **(A)** Initial Presentation: Multiple scattered lesions exhibiting high DWI signal, mild T2-FLAIR hyperintensity, and no enhancement (blue arrows). **(B)** Post-Treatment (3 months after initial therapy): Lesions, particularly in the periventricular regions, show significant reduction in size and signal intensity. **(C)** Disease relapse: Periventricular lesions re-enlarge, exhibiting high DWI signal, persistent T2-FLAIR hyperintensity, and new nodular enhancement (blue arrows). **(D)** Long-term follow-up (7 months after combined corticosteroid and rituximab therapy): Lesions are largely resolved, appearing isointense on DWI, with only residual mild T2 hyperintensity and no enhancement. DWI, Diffusion-Weighted Imaging; MRI, Magnetic Resonance Imaging; T2-FLAIR, T2-weighted Fluid-Attenuated Inversion Recovery.

**Figure 2:**
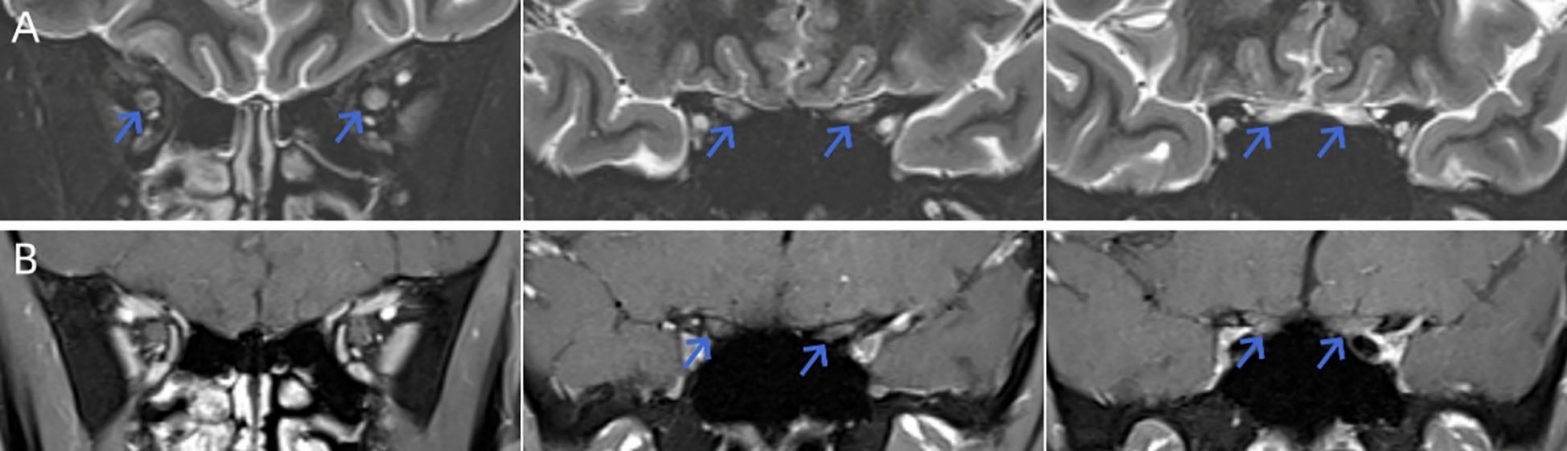
Coronal orbital MRI revealing bilateral optic nerve involvement. **(A)** Coronal fat-suppressed T2-weighted image demonstrates hyperintensity (blue arrows) involving multiple segments of both optic nerves. **(B)** Corresponding coronal contrast-enhanced T1-weighted image shows mild linear enhancement (blue arrows) along the affected nerve segments, consistent with active demyelination. MRI, Magnetic Resonance Imaging.

**Figure 3:**
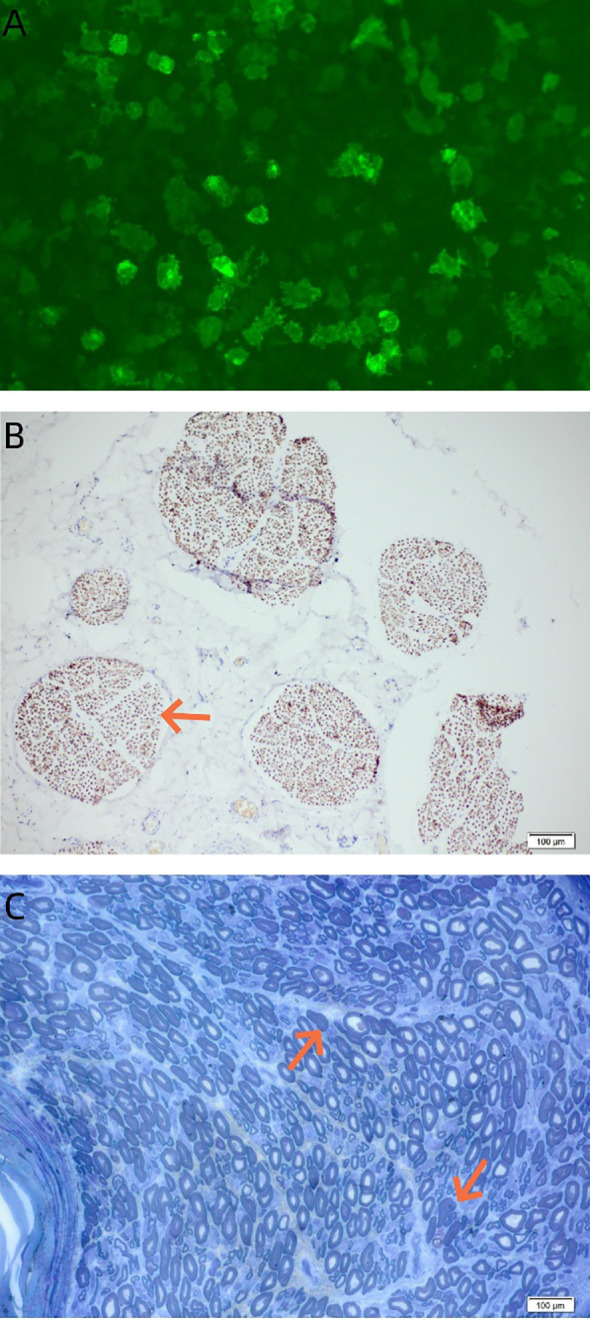
Serological and histopathological features. **(A)** Cell-based assay confirms the presence of serum anti-NF155 IgG4 antibodies (green fluorescence). **(B)** Neurofilament immunostain of sural nerve biopsy reveals a mildly reduced density of small-diameter myelinated fibers in individual nerve fascicles (blue arrows). **(C)** Toluidine blue-stained semi-thin section shows occasional axonal degeneration (blue arrows). anti-NF155, anti-neurofascin 155.

Best-corrected visual acuity was 0.5 in both eyes. Funduscopy showed well-defined optic disc margins with normal coloration bilaterally. Humphrey visual field perimetry was normal (**Supplementary Figure 1B**). However, VEP demonstrated bilaterally prolonged P100 latencies, while optical coherence tomography (OCT) indicated normal retinal nerve fiber layer thickness. Cerebral magnetic resonance imaging (MRI) showed multiple scattered demyelinating lesions (**Figure 1A**). Optic nerve MRI showed the possibility of optic neuritis (**Figures 2A, B**). Repeat electrophysiological studies confirmed a demyelinating sensorimotor polyneuropathy, demonstrating (1): prolonged distal motor latencies (2); reduced motor and sensory nerve conduction velocities (3); prolonged F-wave latencies in the right ulnar nerve; and (4) absent F-waves in multiple nerves of the upper and lower extremities. These findings were consistent with CIDP. Cerebrospinal fluid (CSF) analysis demonstrated albuminocytologic dissociation, featuring (1): markedly elevated protein (344 mg/dL; normal <45 mg/dL) (2); a normal white cell count (0 cells/mm³); and (3) negative oligoclonal IgG bands. Serological testing by fixed cell-based immunofluorescence assay was positive for IgG4 anti-NF155 antibodies (**Figure 3A**).

A right sural nerve biopsy was performed. Histopathological examination revealed evidence of demyelination. Semi-thin sections demonstrated a mildly reduced density of small-diameter myelinated fibers in individual nerve fascicles (**Figure 3B**). Notably, in addition to demyelination, occasional axonal degeneration was observed (**Figure 3C**). These findings—demyelinating features accompanied by mild axonal loss—are consistent with the established pathology of IgG4 anti-NF155 antibody-positive neuropathy, wherein primary antibody-mediated disruption of paranodal axo-glial junctions can lead to demyelination and conduction failure, with axonal injury representing a potential downstream consequence of chronic demyelination. Based on the integration of seropositivity for IgG4 anti-NF155 antibodies, electrodiagnostic confirmation of a demyelinating polyneuropathy meeting CIDP criteria, characteristic CSF albuminocytologic dissociation, and histopathological evidence of demyelination on nerve biopsy, the patient was diagnosed with CCPD.

The patient underwent a gradual taper of oral prednisolone. At the 3-month follow-up, the dose had been reduced to 5 mg/day, and the patient remained clinically stable with no relapses. Follow-up cerebral MRI at 3 months demonstrated significant resolution of the previous demyelinating lesions (**Figure 1B**). The results of CSF analysis (protein 326.10 mg/dL) and electrophysiological studies remained essentially unchanged from previous findings.

However, the patient self-discontinued the 5 mg prednisolone regimen in March 2023 (at the 8-month follow-up). One month after treatment cessation, he experienced a disease relapse manifested as distal limb weakness, reduced muscle strength, and visual field defects, resulting in significant functional impairment.

Re-examination revealed the following (1): a visual field defect in the inner superior quadrant of the right eye (**Supplementary Figure 1C**) (2); enlargement of demyelinating lesions on MRI (**Figure 1C**) (3); persistently elevated CSF protein levels (protein 294.90 mg/dL) (4); worsened peripheral neuropathy on electrophysiological studies, showing mixed sensorimotor involvement (predominantly motor) with both demyelinating and axonal features (predominantly demyelinating); and (5) persistent IgG4 anti-NF155 antibody positivity.

**Figure 4:**
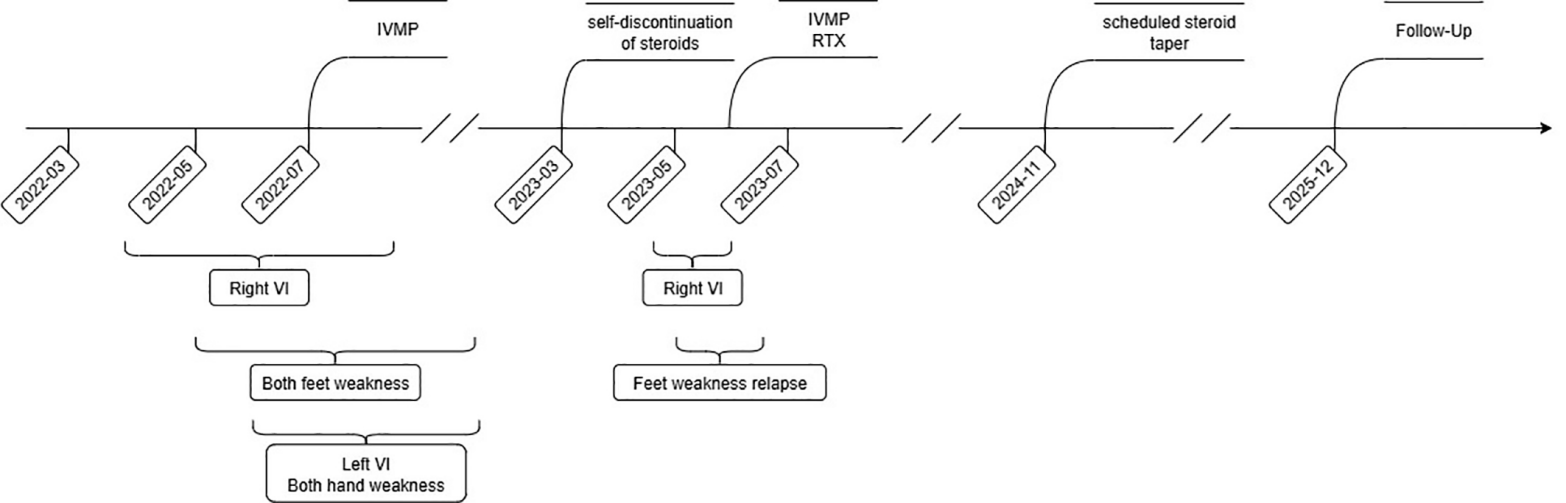
Clinical timeline summarizing disease progression and therapeutic interventions. IVMP, intravenous methylprednisolone; RTX, rituximab; VI, vision impairment.

The patient was treated with intravenous methylprednisolone pulse therapy (1 g/day for 3 days, followed by 500 mg/day for 3 days, and then 250 mg/day for 3 days) to rapidly control acute inflammatory activity. This was followed by a transition to oral prednisolone at an initial dose of 60 mg/day, tapered by 5 mg weekly. Clinical improvement, including complete resolution of visual field defects, was noted within 72 hours (**Supplementary Figure 1D**). Given the diagnosis of IgG4 anti-NF155 antibody-positive CCPD and the recognized poor or limited response to first-line IVIg in this specific subtype (2, 12), B-cell depletion therapy with rituximab was strategically initiated concurrently (100 mg weekly for 4 weeks, as per our institutional protocol). This decision was supported by accumulating evidence demonstrating the efficacy of rituximab in anti-NF155-positive disease, particularly in patients who are steroid-dependent or refractory to conventional therapies (2, 10). Marked improvement in limb weakness was observed at the 1-month follow-up. Post-treatment evaluation demonstrated (1): regression of intracranial demyelinating lesions (**Figure 1D**) (2); reduction of CSF protein from 294.9 mg/dL to 177.7 mg/dL; and (3) significant electrophysiological improvement on nerve conduction studies. The patient remains recurrence-free on regular intravenous rituximab maintenance therapy, aligning with reported outcomes where sustained remission is achievable with B-cell depletion in this antibody-defined phenotype (2, 13). The clinical course is summarized in **Figure 4**.

## 3 Discussion

Herein, we report a biopsy-proven case of IgG4 NF155^+^ CCPD in a young patient who presented with severe optic neuritis as the initial and predominant symptom. With over three years of follow-up, this case is characterized by three notable features (1): initial presentation with isolated optic neuritis (2); diagnostic confirmation via sural nerve biopsy and seropositivity for IgG4 anti-NF155 antibodies; and (3) a clinical course marked by steroid dependence, a relapse following self-discontinuation of steroids, and subsequent sustained remission with corticosteroid pulse therapy and regular rituximab infusion. This case adds to the growing evidence that CCPD, particularly the NF155^+^ subtype, can present with prominent and early visual pathway involvement, and underscores the importance of early antibody testing, histopathological confirmation when indicated, and the strategic use of B-cell depletion therapy for achieving long-term remission.

Historically, symptomatic optic nerve demyelination in CIDP was considered rare and typically associated with advanced disease (14). Recent studies, however, have identified an association with specific autoantibodies. In particular, cohorts of patients with CIDP seropositive for IgG4 anti-NF155 antibodies report a high frequency of optic pathway involvement, which is often subclinical11. Electrophysiological studies using visual evoked potentials (VEPs) have detected prolonged P100 latencies in a substantial proportion of NF155^+^ CIDP/CCPD patients, even among those with preserved visual acuity, suggesting prevalent subclinical optic nerve demyelination in this subgroup (4, 11). This aligns with the broader recognition of CCPD as a phenotype associated with anti-paranodal antibodies, including anti-NF155. Our case, where significant vision loss was the presenting feature, is consistent with the expanding clinical spectrum described in recent literature. For instance, the case series by Kruszewski et al. highlighted that CIDP, including NF155^+^ cases, can present with profound, early visual impairment, thereby challenging the traditional view that isolated optic neuropathy is exceptional in CIDP (9). The underlying pathophysiology in such cases is likely multifactorial. It may involve a direct antibody-mediated effect on central myelin, given the expression of NF155 on oligodendrocytes in the optic nerve. Additionally, in cases presenting with optic disc edema, mechanical effects from elevated intracranial pressure—potentially secondary to impaired cerebrospinal fluid absorption due to markedly elevated protein levels—may contribute to visual loss (9).

The presence of IgG4 anti-NF155 antibodies is associated with a distinct clinicopathological phenotype within the spectrum of inflammatory demyelinating disorders. NF155 is a glial cell adhesion molecule crucial for maintaining the paranodal axo-glial junction in both the central and peripheral nervous systems (8). IgG4 antibodies, which block protein-protein interactions without activating complement, are implicated in the pathogenesis of a “nodo-paranodopathy” that disrupts salutatory conduction (8, 11, 12). The shared expression of NF155 in the CNS and PNS provides a plausible mechanistic basis for the simultaneous or sequential involvement of both compartments, as seen in CCPD. Our case, presenting initially with optic neuropathy followed by classic CIDP features, aligns with and exemplifies this phenotype. This observation is supported by prior cohort studies. For instance, Kawamura et al. and Ogata et al. reported a high frequency of anti-NF155 antibodies in patients diagnosed with CCPD (1, 3). Notably, Ogata et al.’s nationwide survey in Japan further highlighted that optic nerve involvement was a common feature among such antibody-positive CCPD cases (3). Together, these findings reinforce the association between IgG4 anti-NF155 antibodies and a clinical syndrome encompassing both central (e.g., optic neuritis) and peripheral demyelination.

The presentation of isolated or prominent visual loss in a young patient, as seen here, should prompt consideration of CCPD in the differential diagnosis, especially when accompanied by peripheral sensorimotor symptoms or markedly elevated CSF protein. Distinguishing this entity from more common causes of optic disc edema (e.g., idiopathic intracranial hypertension) or optic neuritis-particularly neuromyelitis optica spectrum disorder (NMOSD), myelin oligodendrocyte glycoprotein antibody-associated disease (MOGAD), and multiple sclerosis (MS)—is critical for guiding appropriate investigation and therapy. Key differentiating features include electrodiagnostic evidence of an acquired demyelinating polyneuropathy, IgG4 anti-NF155 seropositivity, markedly elevated CSF protein in the absence of pleocytosis, and the presence of scattered, non-specific CNS demyelinating lesions without the characteristic patterns of MS, AQP4^+^ NMOSD, or MOGAD. A comprehensive comparison of these distinguishing features is provided in **Supplementary Table 1**.

The management of NF155^+^ CCPD with visual involvement necessitates aggressive and timely immunomodulation to prevent irreversible optic nerve damage. The literature suggests that patients with anti-NF155 antibodies often respond poorly or have a limited response to first-line CIDP treatments such as IVIg (15). In contrast, therapies including corticosteroids, rituximab, and plasma exchange have demonstrated efficacy in case series (10). For instance, the two NF155^+^ patients with vision loss in the series by Kruszewski et al. showed significant improvement with corticosteroid-based regimens (9). This underscores the importance of early and accurate diagnosis to guide appropriate therapy. For vision-threatening optic disc edema, surgical interventions such as optic nerve sheath fenestration may be necessary adjuncts, as observed in severe cases (9).

Our study has several limitations that warrant discussion. First, regarding antibody detection methodology, serum anti-NF155 antibodies were identified using a single technique—a cell-based assay (CBA) with human NF155-transfected cells—without confirmation by an alternative method such as enzyme-linked immunosorbent assay (ELISA) using human recombinant NF155 or immunohistochemistry on teased nerve fibers. While CBA is a widely accepted method for detecting anti-nodal/paranodal antibodies, reliance on a single assay increases the risk of false-positive results, particularly if the assay employs tagged recombinant proteins which may elicit antibodies against the tag itself rather than the target antigen. Best practice recommendations, based on studies highlighting discrepancies between assays, advocate for the use of confirmatory techniques (e.g., a combination of CBA and ELISA) to enhance diagnostic specificity. Second, we did not assess anti-NF155 antibodies in the cerebrospinal fluid (CSF). This omission limits our understanding of the intrathecal humoral immune response. Determining the presence and isotype of anti-NF155 antibodies in the CSF could provide critical insights into whether there is local antibody production within the central nervous system compartment, and may help clarify whether intrathecal antibody synthesis contributes to central nervous system involvement, particularly in patients with prominent manifestations such as optic neuritis. Third, the sural nerve biopsy was performed one month after initiation of corticosteroid therapy. This timing may have influenced the histopathological findings, potentially attenuating the degree of demyelination and axonal loss observed. Treatment-related partial remyelination or reduced inflammatory activity could explain the relatively mild changes seen (i.e., mildly reduced density of small-diameter fibers and occasional axonal degeneration), despite the patient’s clinically evident and electrophysiologically confirmed demyelinating polyneuropathy. Therefore, the biopsy findings likely represent post-treatment residual changes rather than the full histopathological spectrum of untreated disease. Fourth, an additional and intriguing observation pertains to the longitudinal antibody titers. Despite clear clinical and radiological improvement following immunosuppressive therapy (corticosteroids and rituximab), the serum IgG4 anti-NF155 antibody titer remained persistently positive and essentially unchanged. Specifically, the titer was 1:100^+^ at initial diagnosis and was again measured at 1:100 (by CBA) during a subsequent disease relapse. This dissociation between clinical activity and antibody level suggests that, in this particular case, the serum IgG4 anti-NF155 antibody titer may not serve as a reliable biomarker for monitoring disease activity or treatment response. This observation raises questions about whether serum autoantibody titers accurately mirror the local immune activity occurring within the nerve parenchyma, and supports the view that clinical assessment, rather than serological titers alone, should guide treatment decisions in this condition.

## 4 Conclusion

In conclusion, our case illustrates that CCPD associated with anti-NF155 antibodies can manifest with severe, early visual impairment as the presenting symptom. This presentation expands the clinical spectrum of NF155^+^ nodo-paranodopathy and highlights the necessity for a high index of suspicion in patients with unexplained optic neuropathy, particularly when combined with peripheral neurological signs. Early recognition, confirmed by specific antibody testing, is paramount for initiating targeted immunotherapies aimed at preserving both visual and neurological function.

## Data Availability

The original contributions presented in the study are included in the article/**Supplementary Material**. Further inquiries can be directed to the corresponding author.
