# Supplementary material for: Case Report: Presenting as optic neuritis—a biopsy-proven IgG4 anti-NF155–positive combined central and peripheral demyelination syndrome
Source: Front Immunol. 2026 May 22;17:1793527. doi: 10.3389/fimmu.2026.1793527 (PMC13236885; doi:10.3389/fimmu.2026.1793527)
Supplement: Supplementary file 1 [file Supplementaryfile1.docx]

Supplementary Material

## Supplementary Figure


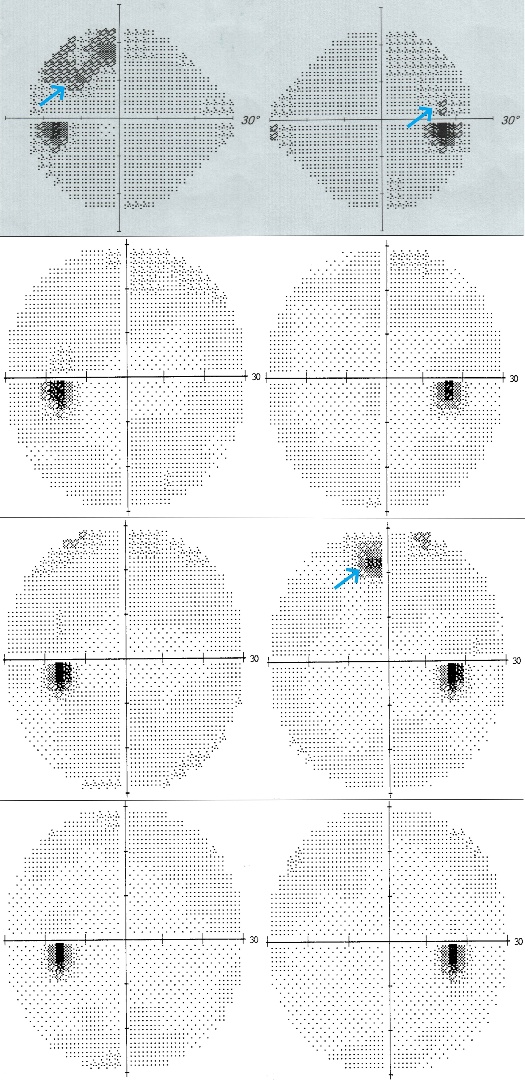


**Supplementary Figure 1. Humphrey visual field perimetry demonstrating the rapid reversibility of quadrantanopia following immunotherapy.**

(A) At initial onset: Bilateral visual field defects are present in the outer superior quadrants (blue arrows).

(B) Three months after initial treatment (corticosteroids): The bilateral outer superior quadrant defects show complete resolution.

(C) At disease relapse (right eye): A visual field defect re-emerges in the inner superior quadrant of the right eye (blue arrows).

(D) Right eye within 72 hours after methylprednisolone pulse therapy: The recurrent inner superior quadrant defect resolves completely, demonstrating rapid therapeutic responsiveness.

**Supplementary Table 1. Key Distinguishing Features of Anti-NF155^+^ CCPD vs. MOGAD, AQP4^+^ NMOSD, and MS.**

| - Feature | - Anti-NF155^+^ CCPD (Present Case) | - MOGAD | - AQP4^+^ NMOSD | - Multiple Sclerosis (MS) |
| --- | --- | --- | --- | --- |
| - Optic neuritis | - Predominantly subclinical (1, 2), rare as an initial or presenting symptom, as in the present case | - Very common (50-65% in adults). Often bilateral, severe, with periorbital pain and optic disc edema (3) | - Common. Often severe, may be unilateral or bilateral. Chiasmal involvement is more common than in MOGAD (4) | - Common. Typically unilateral, mild to moderate severity. Severe optic disc edema/hemorrhage is rare (5) |
| - Myelitis | - May occur (as part of CCPD). Spinal cord lesions may occur but are not the defining feature (6-9) | - Common. Often severe, with longitudinally extensive lesions (LETM), may involve the conus medullaris (3) | - Hallmark feature. Characterized by longitudinally extensive transverse myelitis (LETM). Cord atrophy is common (4) | - Common. Typically short-segment lesions, often peripherally located in the cord (5) |
| - Peripheral Nervous System Involvement | - Hallmark: Demyelinating polyneuropathy fulfilling CIDP criteria, with nerve conduction abnormalities (10, 11) and may have nerve hypertrophy (12) | - Rare/Atypical (3) | - Rare/Atypical (4) | - Absent in typical cases (5) |
| - Optic Nerve MRI | - May show hyperintensity and enhancement, Subclinical involvement is frequent (1, 8) | - Longitudinally extensive (>50% nerve length). Perineural enhancement is characteristic (3) | - Often longitudinally extensive. Isolated chiasmal or optic tract involvement is more suggestive than in MOGAD (4). | - Typically short, focal lesions. Perineural enhancement is uncommon (5). |
| - Brain MRI | - Mostly scattered, non-specific T2/FLAIR hyperintense lesions, which can be distributed in periventricular, subcortical, and infratentorial regions (9, 10) | - "Fluffy," poorly demarcated T2 lesions (ADEM-like). Deep grey matter, brainstem, and cortical lesions can occur. Lesions often resolve (3) | - Lesions favor AQP4-rich areas: periependymal (around 3rd/4th ventricles), hypothalamus, area postrema. "Cloud-like" enhancement (4) | - Well-demarcated, ovoid periventricular, juxtacortical, and infratentorial lesions. Dawson's fingers, central vein sign (5) |
| - CSF Protein Level | - Markedly elevated (often >100 mg/dL, can be >>300 mg/dL), with albuminocytologic dissociation (9, 13) | - Mildly elevated or normal (3) | - Mildly elevated or normal (4) | - Normal or mildly elevated (5) |
| - Oligoclonal Bands | - Usually negative | - Infrequent (<20-30%), often transient if present (3) | - Infrequent (20-30%) (4) | - Extremely frequent (>90%), persistent (5) |
| - Serum Biomarker | - Predominantly IgG4 anti-NF155^+^ (2); IgM anti-NF155 rare (6) | - MOG-IgG antibodies (high titer >1:100 is diagnostic) (3, 4) | - AQP4-IgG antibodies (highly specific) (4) | - No disease-specific serum antibody. Diagnosis relies on clinical/MRI criteria (5) |
| - Treatment Response | IVIg: poor response (2, 10)  Corticosteroids: good initial response, but with dependence (2)   - Rituximab (B-cell depletion): good response (2, 14) | - Excellent response to high-dose corticosteroids. May require chronic immunosuppression for relapsing course (3) | - Requires long-term immunosuppression. Monoclonal antibodies (anti-IL6R, anti-CD19, complement inhibitors) are effective (4) | - Good response to disease-modifying therapies (DMTs) targeting T/B cells and immune trafficking (5) |

CCPD: Combined Central and Peripheral Demyelination, CIDP: Chronic Inflammatory Demyelinating Polyneuropathy, CSF: Cerebrospinal Fluid, IVIg: Intravenous Immunoglobulin, LETM: Longitudinally Extensive Transverse Myelitis, MOGAD: Myelin Oligodendrocyte Glycoprotein Antibody-Associated Disease, MRI: Magnetic Resonance Imaging, MS: Multiple Sclerosis, NMOSD: Neuromyelitis Optica Spectrum Disorders

References

1. Verghese A, Krishnan D, Chia YK, Querol L, Hiew FL. Optic nerve demyelination in igg4 anti-neurofascin 155 antibody-positive combined central and peripheral demyelination syndrome. *Journal of central nervous system disease*. 2021;13:11795735211039913

2. Kira JI. Anti-neurofascin 155 antibody-positive chronic inflammatory demyelinating polyneuropathy/combined central and peripheral demyelination: Strategies for diagnosis and treatment based on the disease mechanism. *Frontiers in neurology.* 2021;12:665136

3. Sechi E, Gastaldi M, Cortese R, Bisecco A, Vogrig A, Giannoccaro MP, et al. Myelin oligodendrocyte glycoprotein antibody-associated disease (mogad): Practical recommendations for diagnosis and management. *Journal of neuroimmunology*. 2026;410

4. Yamout B, Gouider R, Al-Roughani R, Aljarallah S, Shalaby N, Al-Khabouri J, et al. Consensus recommendations for the diagnosis and treatment of neuromyelitis optica spectrum disorders (nmosd): The menactrims guidelines. *CNS Drugs*. 2026;40:283-303

5. Montalban X, Lebrun-Frénay C, Oh J, Arrambide G, Moccia M, Pia Amato M, et al. Diagnosis of multiple sclerosis: 2024 revisions of the mcdonald criteria. *The Lancet. Neurology*. 2025;24:850-865

6. Pegat A, Delmont E, Svahn J, Bernard E, Lessard L, Marignier R, et al. Combined central and peripheral demyelination with igm anti-neurofascin 155 antibodies: Case report. *Neurology(R) neuroimmunology & neuroinflammation*. 2022;9

7. Tajima Y, Matsumura M, Yaguchi H, Mito Y. Possible combined central and peripheral demyelination presenting as optic neuritis, cervical myelitis, and demyelinating polyneuropathy with marked nerve hypertrophy. *Internal medicine*. 2018;57:867-871

8. Kira JI, Yamasaki R, Ogata H. Anti-neurofascin autoantibody and demyelination. *Neurochemistry international.* 2019;130:104360

9. Ogata H, Matsuse D, Yamasaki R, Kawamura N, Matsushita T, Yonekawa T, et al. A nationwide survey of combined central and peripheral demyelination in japan. *Journal of neurology, neurosurgery, and psychiatry.* 2016;87:29-36

10. Hou X, Liang Y, Cui P, Hao J. The clinical features of combined central and peripheral demyelination and antibodies against the node of ranvier. *Multiple sclerosis.* 2022;28:453-462

11. Cortese A, Franciotta D, Alfonsi E, Visigalli N, Zardini E, Diamanti L, et al. Combined central and peripheral demyelination: Clinical features, diagnostic findings, and treatment. *Journal of the neurological sciences.* 2016;363:182-187

12. Ogata H, Zhang X, Inamizu S, Yamashita KI, Yamasaki R, Matsushita T, et al. Optic, trigeminal, and facial neuropathy related to anti-neurofascin 155 antibody. *Annals of clinical and translational neurology*. 2020;7:2297-2309

13. Ogata H, Zhang X, Yamasaki R, Fujii T, Machida A, Morimoto N, et al. Intrathecal cytokine profile in neuropathy with anti-neurofascin 155 antibody. *Annals of clinical and translational neurology.* 2019;6:2304-2316

14. Querol L, Rojas-García R, Diaz-Manera J, Barcena J, Pardo J, Ortega-Moreno A, et al. Rituximab in treatment-resistant cidp with antibodies against paranodal proteins. *Neurology(R) neuroimmunology & neuroinflammation.* 2015;2:e149
